# Islet Transplantation & **β**-Cell Replacement Therapies for Diabetes

**DOI:** 10.1155/2011/157840

**Published:** 2012-05-07

**Authors:** Thierry Berney, Olle Korsgren, Andrew Posselt, Antonello Pileggi

**Affiliations:** ^1^Department of Surgery, Geneva University Hospitals, 1211 Geneva, Switzerland; ^2^Department of Clinical Immunology, Uppsala University Hospital, 75185 Uppsala, Sweden; ^3^Department of Surgery, University of California, San Francisco, CA 94143, USA; ^4^Departments of Surgery, Biomedical Engineering, Microbiology and Immunology, Diabetes Research Institute, University of Miami, Miami, FL 33136, USA

Restoration of physiologic metabolic control is the ultimate therapeutic goal for patients with diabetes but is difficult to achieve by means of medical therapy. Transplantation of islets of Langerhans represents a viable therapeutic option for patients with insulin-dependent diabetes. It is currently offered to patients with a brittle form of diabetes and hypoglycemia unawareness. Current limitations of islet transplantation include scarcity of deceased donor pancreata, partially effective islet isolation and purification protocols, need for life-long systemic immunosuppression, inadequate implantation sites, lack of predictive tests, and noninvasive graft monitoring protocols. 

Great progress has been recorded in different research areas aimed at overcoming current limitations of islet cell transplantation. These areas include stem cells technology, xenotransplantation, development of novel immunosuppressive regimens, immune tolerance, immunoisolation protocols, alternative sites for islet implantation, immune monitoring, and noninvasive imaging techniques. This special issue was open to the scientific community working on the development of beta-cell replacement. Of the several papers received, ten received favorable peer reviews and were selected for publication in this issue to cover specific topics of general interest in this field.

The paper by C. Jahansouz et al. provides a comprehensive and historical overview of beta-cell replacement therapies. The paper by P. Chhabra and K. L. Brayman reviews emerging clinical and preclinical approaches toward enhancing the clinical outcome of islet cell transplantation and includes aspects related to cytoprotection and immune interventions. The paper by H. Wang et al. is focused on important cytoprotective pathways such as carbon monoxide and heme oxygenase-1 and their ability to enhance islet cell viability and engraftment. The paper by X. Wang et al. offers a perspective on the B7-H4 co-stimulation pathway and its potential toward enhancing immune regulation and prolonging islet graft survival.

Recognizing the current limitations of human pancreas donation after brain death, alternative cellular-based approaches are actively being explored to widen the clinical application of beta-cell replacement therapies. The paper by M. R. Hammerman provides interesting new experimental data on porcine islet cell engraftment in xenogeneic recipients without immunosuppression. In his paper H. Noguchi shares an overview of his experience with novel protocols aimed at ameliorating pancreas preservation injury and improving the yield and quality of islets obtained for transplantation. I. Meivar-Levy et al. explore the use of hepatic cells expressing albumin and mesenchymal properties that have been engineered to produce insulin as an alternative and potentially unlimited source of transplantable insulin-producing cells. F. Carlotti et al. assess the important issue of the pros and cons related to the translatability of results obtained in rodent studies to humans when studying beta-cell regeneration.

There are increasing efforts aimed at developing novel implantation sites for islet cells. The paper by D. Espes et al. describes the promising properties of intramuscular site which makes of it a viable option for islet cell implantation.

P. Wang et al. review the important and rapidly evolving field in vivo islet imaging. The use of molecular imaging techniques may be of invaluable assistance toward monitoring islet grafts as well as a tool to better understand the physiopathological changes that occur after transplantation.

*Thierry Berney* *Thierry Berney* *Olle Korsgren* *Olle Korsgren* *Andrew Posselt* *Andrew Posselt* *Antonello Pileggi* *Antonello Pileggi*

